# Microcomputed tomography examination of the cortical and trabecular structures of the cranial cervical spine in dogs and cats

**DOI:** 10.3389/fvets.2026.1888800

**Published:** 2026-07-23

**Authors:** Vanessa Klotz, Anja-Christina Waselau, Yury Zablotski, Andrea Meyer-Lindenberg

**Affiliations:** 1 Clinic for Small Animal Surgery and Reproduction, LMU, Munich, Germany; 2 Clinic for Small Animal Medicine, LMU, Munich, Germany

**Keywords:** atlantoaxial instability, bone parameter, cervical vertebrae, micro-computed tomography, trabecular bone

## Abstract

**Introduction:**

The microarchitectural structure of the cervical spine in small animals, particularly the cranial cervical spine, has received little attention to date. This study aimed to analyze the trabecular and cortical microarchitecture of the first four cervical vertebrae and to identify potential differences between toy- and small-breed dogs and between toy-breed dogs and cats.

**Methods:**

The first four cervical vertebrae from 20 euthanized dogs (10 toy-breed dogs and 10 small-breed dogs) and 20 euthanized cats were examined using micro-computed tomography. The following parameters were analyzed: bone volume fraction (BV/TV), bone surface-to-volume ratio (BS/BV), connectivity density (Conn. D), trabecular number (Tb. N), trabecular thickness (Tb. Th), trabecular separation (Tb. Sp), degree of anisotropy (DA), cortical bone density (mean density), and the dens-to-axis length ratio (DALR).

**Results:**

Trabecular microarchitecture was largely comparable between toy-breed dogs and small-breed dogs; however, cortical bone density was significantly higher in toy-breed dogs than in small-breed dogs. Compared with toy-breed dogs and cats, cats more frequently exhibited a larger BS/BV, a higher DA, and a larger Tb. Sp. In contrast, toy-breed dogs exhibited higher BV/TV, a higher Conn. D, Tb. Th, Tb. N, and mean density. The DALR did not differ significantly among the groups.

**Discussion:**

These findings suggest greater overall stability of the cervical spine in toy-breed dogs compared to cats. Furthermore, these microarchitectural differences may provide useful criteria for selecting appropriate surgical interventions for affected animals.

## 1 Introduction

Lesions of the cervical spine are rare in both dogs and cats (1–3). The most commonly affected vertebrae are C1 and C2 (2, 4), and abnormalities in this region are referred to as atlantoaxial instability (AAI) or atlantoaxial subluxation (AAS). These conditions can be either congenital or acquired (4–6). Acquired, trauma-related fractures, dislocations, or dislocated fractures usually result from car accidents (1, 2, 4). Both congenital and developmental lesions of the cervical spine can predispose animals to AAI (5–10). AAI is primarily observed in toy-breed dogs, but it also rarely occurs in larger dogs and cats (5–7, 11–13). Causes of AAI may include hypoplasia or aplasia of the dens of the axis (8,9,14), malformations of the atlantoaxial ligamentous apparatus (9), incomplete ossification of the neural arch (14–16), or block vertebrae (17).

Both conservative and surgical treatment methods have been described for these conditions (2, 12, 18). For AAI in particular, both dorsal and ventral surgical approaches have been reported (7, 10, 11, 13, 18–24), with ventral fixation frequently employing plates and screws (4, 7, 18, 20, 21, 23). Surgical treatment is particularly challenging in small-breed dogs and cats due to their delicate bone structures (8), as complications such as implant fracture or loosening occur relatively frequently following surgical intervention (10, 19, 21). In human medicine, trabecular structural parameters are known to influence implant stability (25, 26). In contrast, no studies investigating the trabecular and cortical microarchitecture of the cranial cervical spine in dogs and cats have been published in the accessible veterinary literature to date. Therefore, one objective of the present study was to fill this gap and examine the microarchitecture of the first and second cervical vertebrae (C1-C2) compared to the 3rd and 4th cervical vertebrae (C3, C4). In addition, potential differences between toy-breed dogs and small-breed dogs, as well as cats, were evaluated. Furthermore, the size ratio between the dens and the axis was calculated and compared. The results are intended to contribute to a better understanding of the anatomy and, consequently, to the improved selection of implant placement and, thereby, vertebral fixation.

## 2 Materials and methods

### 2.1 Animals and preparation

The cervical spines examined in this study were obtained from 20 dogs and 20 cats weighing between 1.3 and 10.8 kg that died or were euthanized for reasons unrelated to cervical spine disorders at the Clinic for Small Animal Surgery and Reproduction, Ludwig Maximilians University of Munich (LMU). The animal carcasses were donated to the clinic by their owners for scientific purposes. The study was approved by the Ethics Committee of the Faculty of Veterinary Medicine at Ludwig Maximilian University of Munich under file number AZ 348–31-01-2023. Only adult animals were included in the study, and the gender distribution was balanced across all groups to minimize differences in age and sex. The median age was comparable across all groups to minimize age-related differences (Table 1). Animals with diseases that could reduce bone integrity, such as chronic kidney disease (CKD) or hyperadrenocorticism (HAC), were excluded as far as possible based on their medical histories.

**Table 1:** Overview of the study population, including information on animal species, body weight, median age, sex distribution, breed, and absence of trabecular bone.

Group	Small dogs (*n* = 10)	Toy-breed dogs (*n* = 10)	Cats (*n* = 20)
Body weight	5.8–10.8	1.3–5.5	1.9–4.5
Median age (years)	11.25	13	12
Sex	5 M/5 F	5 M/5 F	9 M/11 F
Breeds	Terrier (*n* = 5) Dachshund (*n* = 1) Mixed breeds (*n* = 3) French bulldog (*n* = 1)	Miniature poodle (*n* = 2) Chihuahua (*n* = 2) Miniature pinscher (*n* = 3) Yorkshire terrier (*n* = 3)	European Shorthair (*n* = 15) British Shorthair (*n* = 2) Persian (*n* = 2) Maine Coon mix (*n* = 1)
Absence of trabecular bone in the spinous process, n/N	1/5 (Terriers)	1/2 (Chihuahuas)	

As this was a retrospective study, the final group composition was determined by the availability of specimens fulfilling the predefined inclusion criteria. To allow a more differentiated evaluation of potential body-size-related differences, dogs were further divided into toy- and small-breed groups. Accordingly, the animals were allocated to three groups based on species, breed, and body weight. Group 1 consisted of small-breed dogs (5.8–10.8 kg), Group 2 of toy-breed dogs (1.3–5.5 kg), and Group 3 of cats (1.9–4.5 kg). Groups 1 and 2 each comprised 10 animals, whereas Group 3 comprised 20 animals. Comparisons were performed between Groups 1 and 2 and between Groups 2 and 3. The animal carcasses, which had initially been frozen at −20 °C, were thawed at room temperature. The first four cervical vertebrae (C1, C2, C3, and C4) were removed as a single unit from the head and the remaining cervical spine. A subsequent two-plane radiographic assessment was performed to exclude bone pathology. X-ray examinations were conducted using the Siemens Axiom Luminos dRF X-ray machine (Siemens Healthcare AG, Erlangen, Germany) with the following settings: 50 kV/2 mAs for toy-breed dogs, 50 kV/1.6 mAs for cats, and 55 kV/3 mAs for small-breed dogs. Subsequently, the soft tissue was removed as much as possible, leaving only the ligamentous structures. Only specimens without pathological changes were included. The cervical spines were then stored in 4% formalin until they underwent micro-computed tomography.

Based on the μCT topogram and the implant placement locations described in the literature (5,9,10,21–24), the trabecular bone regions selected for examination were defined as follows: the lateral masses of C1, the spinous process of C2, the dens axis, and the vertebral bodies of the second through fourth cervical vertebrae (C2–C4).

### 2.2 Microcomputed tomography examination

The XtremeCT II *in vivo* scanner (XtremeCT II, Scanco Medical AG, Brüttisellen, Switzerland) was used for the μCT scans of the cervical spine. In this study, an isotropic voxel size of 30.3 μm and 1,000 projections per 180° were selected. The tube voltage was 68 kV, the current was 1,470 μA, and the integration time was 200 ms. Depending on the size of the specimen, the duration of the examination ranged from approximately 30 to 75 min and resulted in 1,440–3,164 image slices per scan.

**Figure 1:**
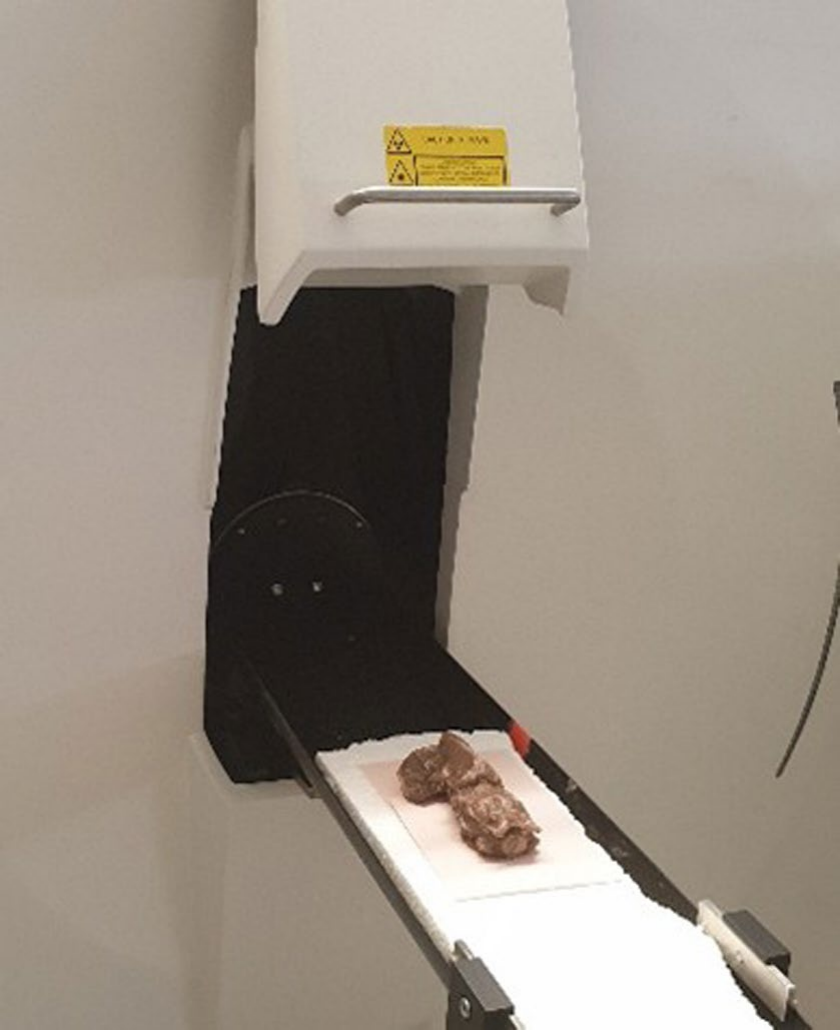
Positioning of the cervical spine (C1–C4) on the µCT table.

For micro-computed tomography imaging, specimens were positioned within the gantry using a custom-made plastic half-shell holder on a homemade polystyrene block, with the dorsal aspect of the vertebrae facing upward and C1 oriented toward the μCT gantry (Figure 1).

### 2.3 Microcomputed tomography analysis

**Figure 2:**
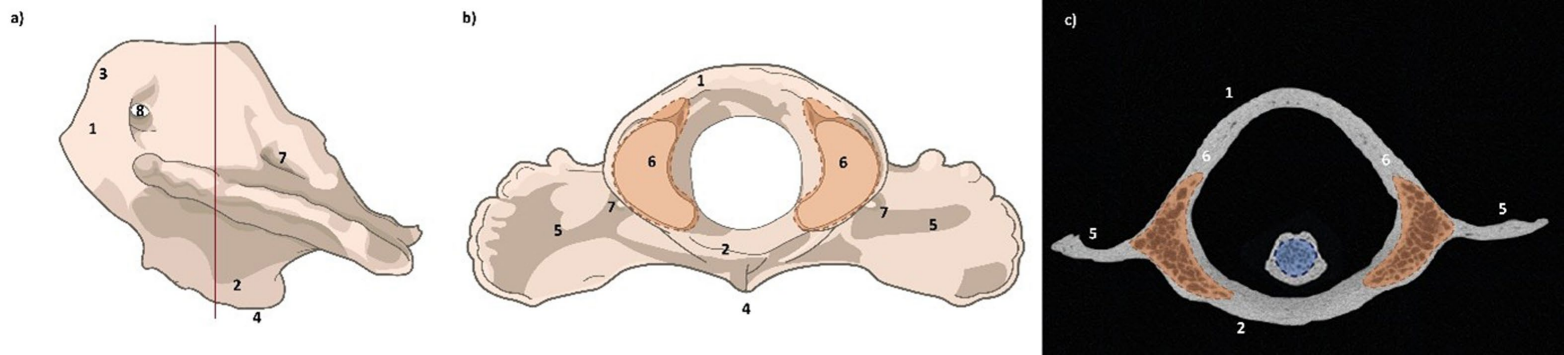
**(A)** Schematic drawing of lateral view of C1. The red line indicates the plane corresponding to cross-sectional image c on µCT imaging. **(B)** Schematic drawing of cranial view of the atlas. **(C)** µCT cross-sectional image of the first cervical vertebra (C1). Shown is the color-coded segmentation of lateral masses (orange) and the dens axis (dark blue). The marked contours indicate the analyzed trabecular bone structures. 1-Dorsal arch, 2-Ventral arch, 3-Dorsal tubercle, 4-Ventral tubercle, 5-Wing of the atlas, 6-Lateral mass, 7-Transverse foramen, 8-Lateral vertebral foramen.

**Figure 3:**
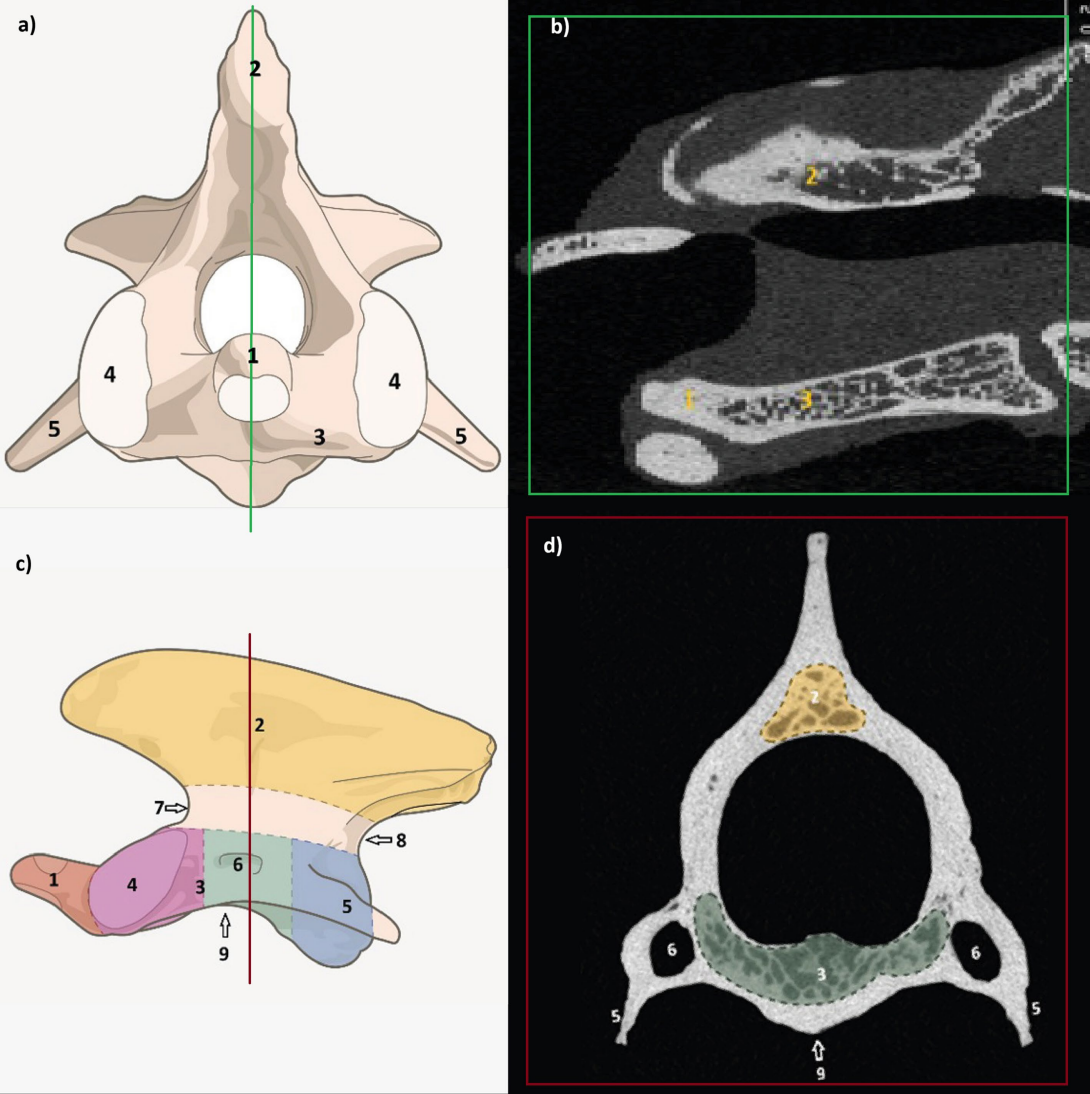
**(A)** Schematic drawing of the axis in cranial view. The green line indicates the plane corresponding to cross-sectional image b on µCT imaging. **(B)** µCT image of the longitudinal section of C2. **(C)** Schematic drawing of the axis in lateral view. The red line indicates the plane corresponding to cross-sectional image d in µCT imaging. **(D)** µCT image of the C2b section in cross-section. The color-coded segmentation highlights the spinous process (yellow), the dens (red), and the defined sections C2A (purple), C2B (green), and C2C (blue). 1-Dens axis, 2-Spinal process, 3-Vertebral body, 4-Anterior articular process, 5-Transverse process, 6-Transverse foramen, 7-Anterior vertebral notch, 8-Posterior vertebral notch, 9-Ventral crest.

**Figure 4:**
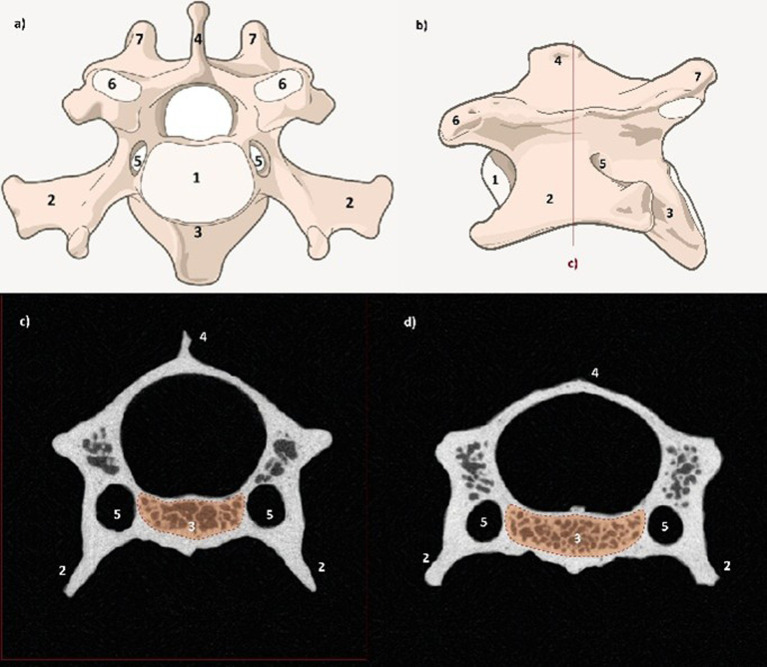
**(A)** Schematic drawing of cranial view of C3 in a dog. **(B)** Drawing of lateral view of C3, representative of C4. The red line indicates the plane corresponding to cross-sectional image c on µCT imaging. **(C)** µCT cross-sectional image with color-coded segmentation of analyzed trabecular bone structures in body of C3 in cross-section. **(D)** µCT cross-sectional image with color-coded segmentation of analyzed trabecular bone structures in body of C4 in cross-section. 1-Cranial end, 2-Transverse process, 3-Vertebral body, 4-Spinal process, 5-Transverse foramen, 6-Cranial articular process, 7-Caudal articular process.

**Figure 5:**
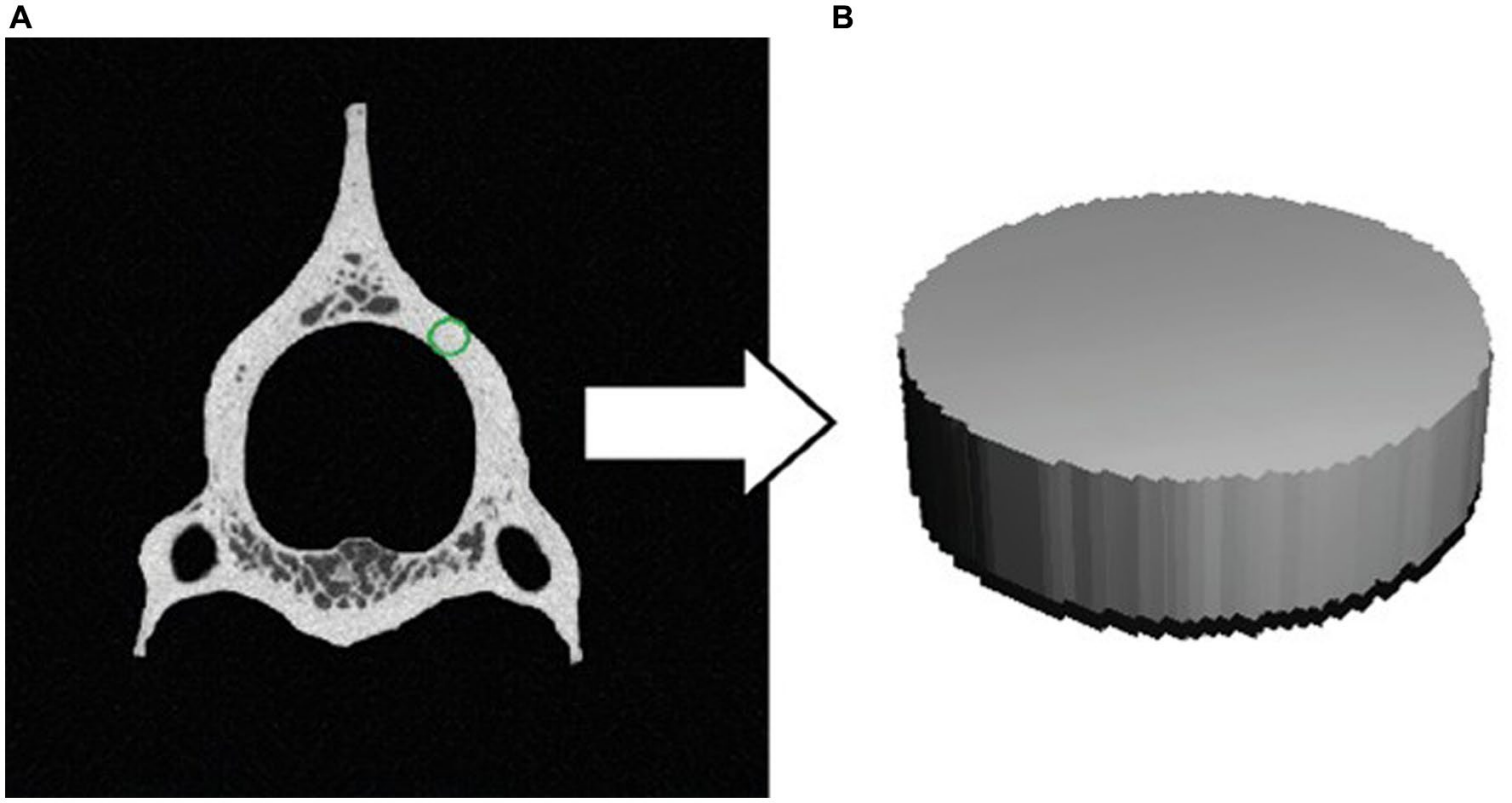
**(A)** µCT cross-sectional image of C2 showing the region of interest (ROI) of the cortical bone in the region of the dorsal vertebral arch lateral to the spinous process, marked in green. **(B)** Three-dimensional reconstruction of cortical bone based on 100 consecutive µCT cross-sectional images.

The μCT analysis software V6.6 (Scanco Medical, Zurich, Switzerland) was used to evaluate the trabecular and cortical bone structure of the cervical vertebrae. The trabecular region of interest (ROI) was manually defined and contoured on every slice (Figures 2–4). This was performed in a cross-sectional view from cranial to caudal. The analysis encompassed the entire trabecular region from the first to the last slice in which at least one trabecular structure was visible. Cortical bone tissue was excluded during the manual contouring. The measured area in C1 was located in the lateral masses on the right and left sides (Figure 2). The dens and the spinous process of the axis were each considered a single contiguous region (Figure 3). The vertebral bodies of C2 (Figure 3), C3, and C4 (Figure 4) were each evenly subdivided into three subregions: a cranial region (a), a middle region (b), and a caudal region (c) (Figure 3). In addition, a region of the dorsal arch of all vertebrae was examined for cortical density. The region encompassed the cortical bone lateral to the spinous process of C2–C4 and, in C1, lateral to the dorsal tubercle, with regions containing cancellous bone excluded. For this purpose, cylindrical ROIs, each 100 scan images in length, were positioned in the middle third of the vertebra (Figure 5).

The thresholds for differentiating between cancellous and cortical bone were determined separately for each animal species and each anatomical location. Measurements were performed manually on each cervical vertebra by three independent observers, and a mean value was calculated. Evaluation of trabecular structural parameters [bone volume fraction BV/TV (%), bone surface-to-volume ratio BS/BV (mm^−1^), connectivity density (Conn. D) (mm^−3^), trabecular number Tb. N (mm^−1^), trabecular thickness Tb. Th (mm), trabecular separation Tb. Sp (mm), and degree of anisotropy (DA)] and the cortical structural parameter mean density (mg HA/ccm) was performed using these established thresholds.

**Figure 6:**
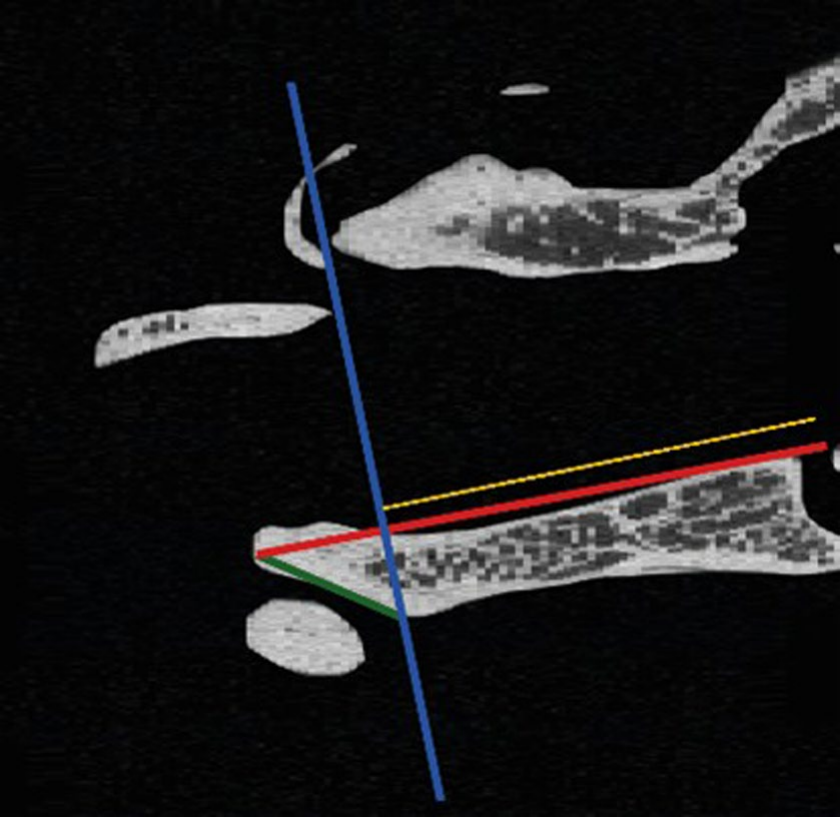
µCT cross-sectional image of the atlas and axis. To determine the length of the dens and the body of the axis, a line was drawn from the tip of the dens to the dorsocaudal aspect of the axis (red). A second line was then drawn perpendicular to this first line through the base of the ventral aspect of the dens (blue). The distance between the tip and the base of the dens was defined as the dens length (green). In the same image, the distance between the intersection of the two lines and the dorsocaudal margin of the axis was defined as the length of the axis body (yellow). The DALR (Dens-to-Axis Length Ratio) was ultimately calculated as the ratio of the dens length to the length of the axis body.

The measurement of the size ratio of the dens-to-axis length ratio (DALR) was performed according to the method of Takahashi et al. (27) (Figure 6). To determine the length of the dens and the body of the axis, a μCT cross-sectional image of the longitudinal section of the axis was evaluated, and a line was first drawn from the tip of the dens to the dorsocaudal aspect of the axis. Subsequently, a second line was drawn perpendicular to this first line through the lower edge of the ventral side of the dens. The distance between the tip and the base of the dens was defined as the dens length. The distance between the intersection of the two lines and the dorsocaudal margin of the axis was defined as the axis body length. The DALR was calculated as the ratio of dens length to axis body length.

### 2.4 Statistical analysis

Regression models were used to examine differences based on breed (toy-breed dogs and small-breed dogs) as well as between the toy-breed dogs and cats within each spinal segement. The following parameters were analyzed: BV/TV, BS/BV, Conn.D, DA, Tb. Sp, Tb. N, Tb. Th, and mean density. In addition, the DALR was compared across dog breeds and between toy-breed dogs and cats. The following model assumptions were always tested: [1] normal distribution of the underlying population of the sample using the Shapiro–Wilk test; [2] homogeneity of variances between groups using the Bartlett test; [3] and homoscedasticity using the Breusch–Pagan test. If these assumptions were met, the standard linear models (ordinary least squares) or linear mixed models (for repeated measurements) were subsequently applied. To evaluate the DALR, linear regression models were used in both cases to analyze differences between dog breeds as well as between toy-breed dogs and cats. If the assumptions were not met, a robust linear mixed regression was applied. All contrasts between specific groups were evaluated after model fitting using estimated marginal means (R package emmeans), without Tukey’s correction for multiple comparisons. Results with a *p*-value < 0.05 were considered statistically significant, whereas results with *p*-values between 0.05 and 0.1 were considered trends. Statistical analysis was performed using R software version 4.5.0 (11 April 2025).

## 3 Results

### 3.1 Comparisons were performed between small- and toy-breed dogs, as well as between cats and toy-breed dogs

**Figure 7:**
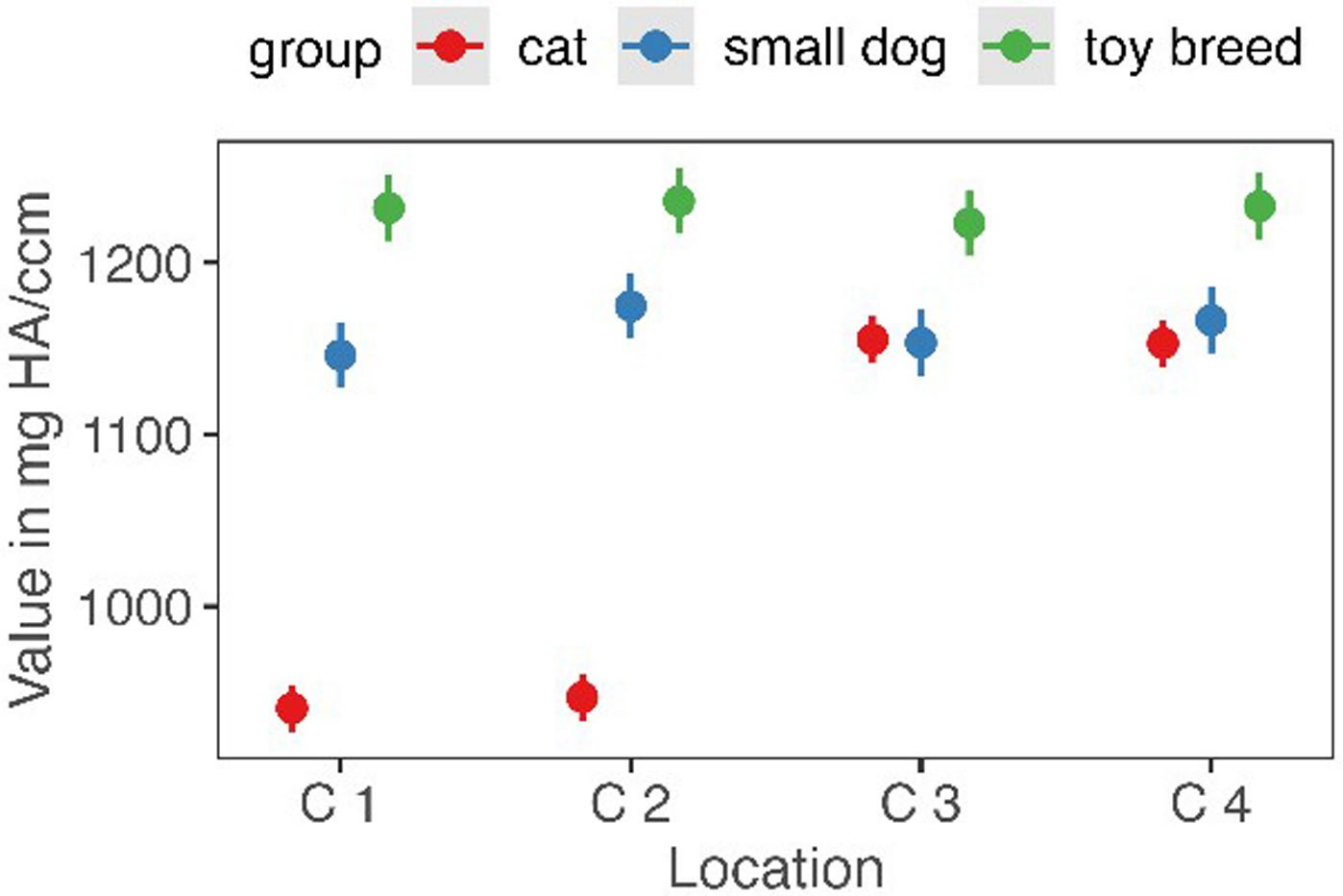
Comparison of cortical bone density in the dorsal vertebral arch lateral to the spinous process or dorsale tubercle of C1-C4 between cats (*n* = 20), small dogs (*n* = 10) and toy breeds (*n* = 10).

Among the dogs examined (Table 1), one animal per group lacked measurable trabecular structure in the spinous process. The affected dogs were a Chihuahua from Group 1 and a Parson Russell Terrier from Group 2. Apart from this, all vertebral segments from C1 to C4 were included in the analysis. The comparison of the right and left sides of C1 showed no significant differences between the groups in the trabecular analysis (*p* ≥ 0.5056), which is why they were analyzed together. The results of the measurements for the individual vertebrae and groups are listed in Tables 2–6, Supplementary Tables S1–S2, and Figure 7. The size ratio of the dens to the axis showed no significant differences between toy-breed dogs and small-breed dogs (*p* = 0.7269) or between toy-breed dogs and cats (*p* = 0.2188).

**Table 2:** Median values and corresponding confidence intervals of bone microarchitecture parameters in cervical vertebra C1 stratified by breed and animal species.

Vertebra	Parameter	Small-breed dogs (*n* = 10)	Toy-breed dogs (*n* = 10)
C1	BV/TV (%)	0.412 (0.320–0.503)	0.361 (0.269–0.453)
	BS/BV (mm^−1^)	13.03 (11.08–15.0)	14.79 (12.85–16.7)
	Conn. D (mm^−3^)	13.04 (10.57–15.5)	14.52 (12.05–17.0)
	DA (dimensionless)	1.18 (1.14–1.21)	1.19 (1.16–1.23)
	Tb. Sp (mm)	0.245 (0.1891–0.301)	0.255 (0.1992–0.311)
	Tb. N (mm^−1^)	2.53 (2.33–2.73)	2.62 (2.42–2.82)
	Tb. Th (mm)	0.159 (0.1333–0.185)	0.137 (0.1116–0.163)
Vertebra	Parameter	Toy-breed dogs (*n* = 10)	Cats (*n* = 20)
C1	BV/TV (%)	0.388 (0.3343–0.442)	0.388 (0.3345–0.442)
	BS/BV (mm^−1^)	13.83 (12.59–15.1)	12.51 (11.27–13.8)
	Conn. D (mm^−3^)	13.93 (12.37–15.49)*	8.47 (6.91–10.03)*
	DA (dimensionless)	1.18 (1.16–1.21)†	1.15 (1.12–1.17)†
	Tb. Sp (mm)	0.2505 (0.2105–0.290)	0.2701 (0.2302–0.310)
	Tb. N (mm^−1^)	2.57 (2.43–2.72)*	2.34 (2.19–2.48)*
	Tb. Th (mm)	0.149 (0.1338–0.163)	0.163 (0.1481–0.178)

BV/TV, bone volume fraction; BS/BV, bone surface to volume ratio; Conn. D, connectivity density; DA, degree of anisotropy; Tb. Sp, trabecular separation; Tb. N, trabecular number; Tb. Th, trabecular thickness. Comparisons were performed between small dogs and toy-breed dogs, and toy-breed dogs and cats. Significant differences (*p* < 0.05) are indicated by an asterisk (*), trends (*p* = 0.05–0.1) by a dagger (†).

**Table 3:** Median values and corresponding confidence intervals of bone microarchitecture parameters in cervical vertebra C3, subdivided by anatomical region, stratified by breed.

C3	Parameter	Small-breed dogs (*n* = 10)	Toy-breed dogs (*n* = 10)
C3A	BV/TV (%)	0.538 (0.441–0.635)	0.494 (0.397–0.591)
	BS/BV (mm^−1^)	10.06 (8.01–12.1)	12.06 (10.01–14.1)
	Conn. D (mm^−3^)	10.66 (7.52–13.8)†	14.41 (11.27–17.5)†
	DA (dimensionless)	1.17 (1.12–1.22)	1.21 (1.16–1.26)
	Tb. Sp (mm)	0.177 (0.1154–0.238)	0.172 (0.1105–0.233)
	Tb. N (mm^−1^)	2.58 (2.33–2.82)	2.79 (2.55–3.03)
	Tb. Th (mm)	0.208 (0.1806–0.236)†	0.172 (0.1447–0.200)†
C3B	BV/TV (%)	0.409 (0.312–0.506)	0.330 (0.233–0.427)
	BS/BV (mm^−1^)	12.16 (10.11–14.2)	14.44 (12.39–16.5)
	Conn. D (mm^−3^)	9.75 (6.61–12.9)	12.02 (8.88–15.2)
	DA (dimensionless)	1.15 (1.11–1.20)	1.20 (1.15–1.25)
	Tb. Sp (mm)	0.254 (0.1928–0.316)	0.306 (0.2448–0.368)
	Tb. N (mm^−1^)	2.36 (2.12–2.60)	2.31 (2.07–2.56)
	Tb. Th (mm)	0.171 (0.1430–0.198)	0.139 (0.1116–0.167)
C3C	BV/TV (%)	0.551 (0.454–0.648)	0.489 (0.392–0.586)
	BS/BV (mm^−1^)	10.58 (8.53–12.6)	12.47 (10.42–14.5)
	Conn. D (mm^−3^)	13.82 (10.69–17.0)*	18.61 (15.48–21.7)*
	DA (dimensionless)	1.14 (1.09–1.19)	1.18 (1.13–1.23)
	Tb. Sp (mm)	0.155 (0.0940–0.217)	0.167 (0.1054–0.228)
	Tb. N (mm^−1^)	2.79 (2.55–3.04)	2.94 (2.70–3.18)
	Tb. Th (mm)	0.198 (0.1704–0.225)†	0.165 (0.1373–0.192)†

BV/TV, bone volume fraction; BS/BV, bone surface to volume ratio; Conn. D, connectivity density; DA, degree of anisotropy; Tb. Sp, trabecular separation; Tb. N, trabecular number; Tb. Th, trabecular thickness. Regions: cranial corpus (C3A), middle corpus (C3B), caudal corpus (C3C). Comparisons were made between small-breed dogs and toy-breed dogs. Significant differences (*p* < 0.05) are indicated by an asterisk (*), trends (*p* = 0.05–0.1) by a dagger (†).

**Table 4:** Median values and corresponding confidence intervals of bone microarchitecture parameters in cervical vertebra C4, subdivided by anatomical region, stratified by breed.

C4	Parameter	Small-breed dogs (*n* = 10)	Toy-breed dogs (*n* = 10)
C4A	BV/TV (%)	0.517 (0.420–0.614)	0.439 (0.342–0.536)
	BS/BV (mm^−1^)	10.53 (8.48–12.6)†	13.05 (11.0–15.1)†
	Conn. D (mm^−3^)	12.20 (9.06–15.3)*	17.36 (14.22.–20.5)*
	DA (dimensionless)	1.19 (1.14–1.23)	1.23 (1.18–1.28)
	Tb. Sp (mm)	0.182 (0.1209–0.244)	0.202 (0.1403–0.263)
	Tb. N (mm^−1^)	2.62 (2.38–2.86)	2.74 (2.50–2.98)
	Tb. Th (mm)	0.197 (0.1692–0.224)*	0.157 (0.1296–0.185)*
C4B	BV/TV (%)	0.444 (0.347–0.541)	0.396 (0.299–0.493)
	BS/BV (mm^−1^)	11.77 (9.72–13.8)	13.44 (11.39–15.5)
	Conn. D (mm^−3^)	10.38 (7.24–13.5)	13.96 (10.82–17.1)
	DA (dimensionless)	1.10 (1.06–1.15)*	1.18 (1.14–1.23)*
	Tb. Sp (mm)	0.226 (0.1651–0.288)	0.237 (0.1758–0.299)
	Tb. N (mm^−1^)	2.45 (2.21–2.70)	2.58 (2.34–2.82)
	Tb. Th (mm)	0.179 (0.1512–0.206)	0.150 (0.1223–0.177)
C4C	BV/TV (%)	0.547 (0.450–0.644)	0.455 (0.358–0.552)
	BS/BV (mm^−1^)	11.08 (9.04–13.1)†	13.75 (11.71–15.8)†
	Conn. D (mm^−3^)	15.12 (11.99–18.3)*	21.56 (18.42–24.7)*
	DA (dimensionless)	1.16 (1.12–1.21)	1.18 (1.14–1.23)
	Tb. Sp (mm)	0.155 (0.0936–0.216)	0.167 (0.1060–0.229)
	Tb. N (mm^−1^)	2.85 (2.60–3.09)	2.97 (2.73–3.21)
	Tb. Th (mm)	0.189 (0.1614–0.216)†	0.155 (0.1278–0.183)†

BV/TV, bone volume fraction; BS/BV, bone surface to volume ratio; Conn. D, connectivity density; DA, degree of anisotropy; Tb. Sp, trabecular separation; Tb. N, trabecular number; Tb. Th, trabecular thickness. Regions: cranial corpus (C4A), middle corpus (C4B), caudal corpus (C4C). Comparisons were performed between small-breed dogs and toy-breed dogs. Significant differences (*p* < 0.05) are indicated by an asterisk (*), trends (*p* = 0.05–0.1) by a dagger (†).

**Table 5:** Median values and corresponding confidence intervals of bone microarchitecture parameters in cervical vertebra C3, subdivided by anatomical region, stratified by animal species.

C3	Parameter	Toy-breed dogs (*n* = 10)	Cats (*n* = 20)
C3A	BV/TV (%)	0.519 (0.4624–0.576)*	0.361 (0.3040–0.418)*
	BS/BV (mm^−1^)	10.96 (9.64–12.3)*	13.75 (12.42–15.1)*
	Conn. D (mm^−3^)	12.33 (10.39–14.26)†	12.74 (10.81–14.67)
	DA (dimensionless)	1.19 (1.15–1.23)*	1.35 (1.32–1.40)*
	Tb. Sp (mm)	0.1764 (0.1327–0.220)*	0.2544 (0.2107–0.298)*
	Tb. N (mm^−1^)	2.65 (2.48–2.82)	2.48 (2.31–2.65)
	Tb. Th (mm)	0.190 (0.1744–0.206)*†	0.148 (0.1324–0.164)*
C3B	BV/TV (%)	0.375 (0.3177–0.431)*	0.273(0.2158–0.330)*
	BS/BV (mm^−1^)	13.22 (11.89–14.5)*	15.59 (14.26–16.9)*
	Conn. D (mm^−3^)	10.62 (8.68–12.55)	9.26 (7.33–11.20)
	DA (dimensionless)	1.18 (1.14–1.22)*	1.49 (1.45–1.52)*
	Tb. Sp (mm)	0.2778 (0.2341–0.322)*	0.3561 (0.3124–0.400)*
	Tb. N (mm^−1^)	2.34 (2.17–2.50)*	2.06 (1.89–2.23)*
	Tb. Th (mm)	0.155 (0.1389–0.171)*	0.131 (0.1149–0.147)*
C3C	BV/TV (%)	0.524 (0.4672–0.581)*	0.381 (0.3245–0.438)*
	BS/BV (mm^−1^)	11.43 (10.10–12.8)*	14.98 (13.65–16.3)*
	Conn. D (mm^−3^)	15.47 (13.54–17.40)*†	18.10 (16.17–20.03)†
	DA (dimensionless)	1.16 (1.12–1.20)*	1.27 (1.23–1.31)*
	Tb. Sp (mm)	0.1615 (0.1178–0.205)†	0.2207 (0.1770–0.264)†
	Tb. N (mm^−1^)	2.85 (2.69–3.02)	2.76 (2.59–2.93)
	Tb. Th (mm)	0.181 (0.1652–0.197)*†	0.138 (0.1224–0.154)*

BV/TV, bone volume fraction; BS/BV, bone surface to volume ratio; Conn. D, connectivity density; DA, degree of anisotropy; Tb. Sp, trabecular separation; Tb. N, trabecular number; Tb. Th, trabecular thickness. Regions: cranial corpus (C3A), middle corpus (C3B), caudal corpus (C3C). Comparisons were made between toy-breed dogs and cats. Significant differences (*p* < 0.05) are indicated by an asterisk (*), trends (*p* = 0.05–0.1) by a dagger (†).

**Table 6:** Median values and corresponding confidence intervals of bone microarchitecture parameters in cervical vertebra C4, subdivided by anatomical region, stratified by animal species.

C4	Parameter	Toy-breed dogs (*n* = 10)	Cats (*n* = 20)
C4A	BV/TV (%)	0.483 (0.4265–0.540)*	0.306 (0.2488–0.363)*
	BS/BV (mm^−1^)	11.66 (10.33–13.0)*†	15.45 (14.12–16.8)*
	Conn. D (mm^−3^)	14.25 (12.32–16.18)*	12.45 (10.52–14.38)
	DA (dimensionless)	1.21 (1.17–1.24)*	1.32 (1.28–1.35)*
	Tb. Sp (mm)	0.1922 (0.1485–0.236)*	0.3100 (0.2662–0.354)*
	Tb. N (mm-1)	2.67 (2.51–2.84)*	2.27 (2.10–2.44)*
	Tb. Th (mm)	0.176 (0.1605–0.192)*	0.131 (0.1151–0.147)*
C4B	BV/TV (%)	0.425 (0.3683–0.482)*	0.233 (0.1757–0.289)*
	BS/BV (mm-1)	12.52 (11.19–13.8)*	17.23 (15.90–18.6)*
	Conn. D (mm-3)	11.83 (9.90–13.76)	9.75 (7.81–11.68)
	DA (dimensionless)	1.14 (1.10–1.18)*	1.34 (1.30–1.38)*
	Tb. Sp (mm)	0.2319 (0.1882–0.276)*	0.4147 (0.3710–0.458)*
	Tb. N (mm-1)	2.51 (2.34–2.68)*	1.92 (1.76–2.09)*
	Tb. Th (mm)	0.164 (0.1484–0.180)*	0.118 (0.1026–0.134)*
C4C	BV/TV (%)	0.504 (0.4467–0.560)	0.482 (0.4254–0.539)
	BS/BV (mm^−1^)	12.31 (10.99–13.6)†	12.19 (10.87–13.5)
	Conn. D (mm^−3^)	17.85 (15.91–19.78)*	14.83 (12.90–16.76)*
	DA (dimensionless)	1.17 (1.14–1.21)	1.26 (1.22–1.29)*
	Tb. Sp (mm)	0.1610 (0.1173–0.205)	0.1749 (0.1312–0.219)
	Tb. N (mm^−1^)	2.90 (2.73–3.07)	2.91 (2.74–3.08)
	Tb. Th (mm)	0.171 (0.1556–0.187)†	0.168 (0.1520–0.184)

BV/TV, bone volume fraction; BS/BV, bone surface-to-volume ratio; Conn. D, connectivity density; DA, degree of anisotropy; Tb. Sp, trabecular separation; Tb. N, trabecular number; Tb. Th, trabecular thickness. Regions: cranial corpus (C4A), middle corpus (C4B), caudal corpus (C4C). Comparisons were performed between toy-breed dogs and cats. Significant differences (*p* < 0.05) are indicated by an asterisk (*), trends (*p* = 0.05–0.1) by a dagger (†).

### 3.2 Differences in the microarchitecture of toy-breed dogs and small-breed dogs

For vertebral bodies C2 through C4, region-specific differences in trabecular bone parameters were observed within each vertebra, and these patterns were consistent across the evaluated groups. In C2, BV/TV, Tb. N, and Tb. Th were lowest in region A. In contrast, Tb. Sp, Conn. D, and BS/BV showed their lowest values in region B, whereas DA was lowest in region C.

A comparable distribution pattern was observed in C3 and C4. In these vertebrae, BS/BV was lowest in region A, while BV/TV, Tb. N, Tb. Th, and Conn. D reached their lowest values in region B, and Tb. Sp was lowest in region C. Across all examined vertebrae, only isolated differences in trabecular bone parameters were found among the groups, and no consistent pattern of intergroup differences could be identified. Nevertheless, toy-breed dogs consistently exhibited higher mean cortical density.

### 3.3 Differences in trabecular and cortical structural parameters of C1

No significant differences in trabecular microarchitecture were observed between the groups for the C1 vertebral body (Table 2). Toy-breed dogs exhibited a significantly higher cortical bone density (*p* < 0.0001) (Figure 7).

### 3.4 Differences in trabecular and cortical structural parameters of C2

At C2 (Supplementary Table S1), most differences occurred in the dens. The BV/TV (*p* = 0.0327) and Tb. Th (*p* = 0.0103) were significantly higher in small-breed dogs, and BS/BV (*p* = 0.0340) as well as Conn. D (*p* = 0.0051) were significantly lower compared with toy-breed dogs. At the spinous process, Tb. Th (*p* = 0.0477) was significantly lower in toy-breed dogs, and at C2A, Tb. Sp (*p* = 0.0357) was significantly higher in toy-breed dogs. Toy-breed dogs also showed significantly higher cortical bone density at C2 (*p* < 0.0001).

### 3.5 Differences in the trabecular and cortical structural parameters of C3

In the C3C region (Table 3), Conn. D (*p* = 0.0349) was significantly higher in toy-breed dogs than in small-breed dogs. Similarly, cortical bone density was significantly higher (p < 0.0001).

### 3.6 Differences in trabecular and cortical structural parameters of C4

In the C4A region (Table 4), Tb. Th (*p* = 0.0466) was significantly higher in small-breed dogs and Conn. D (*p* = 0.0233) was lower. In the C4B region, DA (*p* = 0.0211) was significantly higher in toy-breed dogs, as is Conn. D (*p* = 0.0049) at C4C and cortical bone density (p < 0.0001) across the entire C4.

### 3.7 Differences in the microarchitecture of toy-breed dogs and cats

In terms of spatial distribution within the C2–C4 vertebrae, cats, in contrast to toy-breed dogs, exhibited a uniform pattern with the lowest values for BV/TV, Conn. D, Tb. N, and Tb. Th in region B. For BS/BV, DA, and Tb. Sp, the lowest values predominantly occurred in the caudal regions of the vertebral bodies, with only minor shifts in values between C3 and C4.

Analysis of the trabecular and cortical microarchitecture revealed consistent group-specific differences between toy-breed dogs and cats across all examined locations of the C1 to C4 vertebrae (Tables 2, 4–6, Supplementary Table S2). Overall, toy-breed dogs predominantly exhibited higher values for BV/TV, Tb. N, Tb. Th, and mean density, whereas cats showed higher values for BS/BV, DA, and Tb. Sp. Conn. D did not show a consistent group-specific pattern and varied locally.

### 3.8 Differences in trabecular and cortical structural parameters of C1

In the C1 region (Table 2), Conn. D (*p* < 0.0001) and Tb. N (*p* = 0.0254) were significantly higher in toy-breed dogs compared to cats. Toy-breed dogs also exhibited significantly higher bone density (*p* < 0.0001) (Figure 7).

### 3.9 Differences in trabecular and cortical structural parameters of C2

Within the dens, cats exhibited significantly higher BV/TV (*p* = 0.0070), DA, and Tb. N (p < 0.0001), and lower Conn. D (*p* = 0.0150). At the spinous process, however, BS/BV and Tb. Sp (*p* < 0.0001) were higher in cats, while BV/TV (p < 0.0001), Conn. D (*p* = 0.0067), Tb. N, and Tb. Th (p < 0.0001) were higher in toy-breed dogs. Within C2A (Supplementary Table S2), toy-breed dogs had significantly higher Tb. Sp (*p* = 0.0428) and Tb. N

(*p* = 0.0198). In the C2B region, toy-breed dogs also had significantly higher BV/TV, Tb. N, and Tb. Th (*p* < 0.0001). Cats showed significantly higher BS/BV and Tb. Sp (p < 0.0001). In the C2C segment, BS/BV (*p* = 0.0016) was higher in cats, while BV/TV (*p* = 0.0281), DA (p < 0.0001), and Tb. Th

(*p* = 0.0173) were lower. Toy-breed dogs exhibited significantly higher bone density at C2 (*p* < 0.0001) (Figure 7).

### 3.10 Differences in trabecular and cortical structural parameters of C3

At C3A and C3B (Table 5), cats consistently showed higher values for BS/BV, DA, and Tb. Sp (*p* ≤ 0.0135) as well as lower values for BV/TV and Tb. Th (*p* ≤ 0.0002). In addition, cats at C3B showed a lower Tb. N (*p* = 0.0246). At C3C, cats again had higher BS/BV (*p* = 0.0002) and DA (p < 0.0001), as well as lower BV/TV (*p* = 0.0005) and Tb. Th (p = 0.0002). Cats exhibited significantly lower cortical bone density in C3 than toy-breed dogs (p < 0.0001).

### 3.11 Differences in trabecular and cortical structural parameters of C4

At C4A and C4B (Table 6), cats consistently showed higher values for BS/BV, DA, and Tb. Sp (p ≤ 0.0002), as well as lower values for BV/TV, Tb. N, and Tb. Th (*p* ≤ 0.0009). At C4C, cats exhibited higher DA (*p* = 0.0027) and lower Conn. D (*p* = 0.0305). Toy-breed dogs again showed significantly higher bone density (p < 0.0001) (Figure 7).

## 4 Discussion

The present study provides a comparative analysis of the trabecular microarchitecture of the cranial cervical spine in cats and dogs. Comparisons were performed between toy-breed dogs and small-breed dogs, as well as between cats and toy-breed dogs. Overall, cats exhibited significantly higher values for BS/BV and Tb. Sp, whereas dogs, particularly toy-breed dogs, showed higher values for Tb. N, BV/TV, Tb. Th, and mean density. As only minor differences were observed among the investigated dog breeds, their trabecular microarchitecture appears to be largely comparable, suggesting similar biomechanical properties. In contrast, the higher BS/BV and Tb. Sp values observed in cats indicate a more porous and anisotropic trabecular structure, which may be associated with reduced mechanical stability than in dogs. The biomechanical implications of these findings are discussed below.

The upper cervical spine is essential for the stability and mobility of the craniocervical junction. Conditions such as AAI or fractures in this region present significant surgical challenges, particularly in small-animal patients (8). An understanding of bone microarchitecture is important for implant placement and surgical stabilization. Analysis of trabecular microstructure in different regions of the canine cervical spine provides insight into its mechanical load-bearing capacity and potential structural weak points (28–32). The BV/TV ratio is an indicator of bone breaking load (33) and exerts a decisive influence on the mechanical stability of trabecular bone (29, 30, 32). Lower BV/TV values are associated with a higher incidence of microscopic bone damage (28), whereas higher values reflect improved primary stability of implants (34). In addition, BV/TV correlates with Tb. N and Tb. Sp. (28, 32, 34). In the present study, only minor differences in these parameters were observed between toy-breed dogs and small-breed dogs. A significant increase in BV/TV was detected solely in the dens of small-breed dogs. In contrast, BV/TV was significantly higher in toy-breed dogs than in cats in several regions. These results indicate a potentially reduced stability of the dens in toy-breed dogs. Furthermore, they suggest that the examined segments of the cervical spine exhibit greater stability in dogs than in cats.

BS/BV describes the trabecular bone surface area relative to the trabecular bone volume (36). In the present study, cats exhibited significantly higher BS/BV values. Because BS/BV is negatively correlated with BV/TV (35), this finding may indicate lower trabecular structural integrity in cats than in toy-breed dogs.

Conn. D reflects the degree of trabecular connectivity, although it does not provide information on the quality of individual trabecular connections (36). It is positively correlated with both Tb. N and BV/TV (36). In the present study, Conn. D was occasionally lower in small-breed dogs than in toy-breed dogs. In addition, several regions showed higher Conn. D values in toy-breed dogs than in cats, suggesting more pronounced trabecular interconnection in toy-breed dogs within the study population.

DA represents the spatial orientation of the trabeculae within the bone and is negatively correlated with bone density (37). Failure load is partly influenced by microdamage within trabecular bone, which is associated, among other factors, with reduced BV/TV and increased DA (30). In the present study, only minor differences in DA were observed between the evaluated dog breeds. Cats occasionally exhibited higher DA values than dogs. However, no consistent intergroup pattern was identified.

Higher Tb. N (28) and greater Tb. Th (38) are generally associated with increased bone strength and contribute to implant stability (34). In contrast, increased Tb. Sp is related to reduced structural integrity of the bone (39). In the present study, trabecular parameters were overall comparable across the evaluated dog breeds. Within the dens, toy-breed dogs showed higher Tb. Sp than small-breed dogs, whereas Tb. Th was higher in larger breeds, which may indicate reduced structural robustness of the dens in toy-breed dogs. Compared with cats, toy-breed dogs occasionally showed higher Tb.N and lower Tb.Sp values. In addition, Tb. Th was frequently greater in toy-breed dogs. Overall, these findings suggest a more favorable trabecular microarchitecture in toy-breed dogs than in cats within the present study population.

In addition to trabecular bone parameters, the cortical density of the vertebrae was also assessed. The cortex plays a crucial role in bone stability and fracture resistance (40, 41) as well as in implant retention (42). Incomplete ossification may further contribute to instability by causing joint laxity, particularly in combination with structural deficits of the atlantoaxial ligaments or their osseous attachment sites (16, 43). In dogs with incomplete ossification, significantly lower bone density of the neural arch has been reported compared with unaffected dogs (44). In the present study, cortical density showed a consistent pattern across the examined vertebrae, with toy-breed dogs exhibiting significantly higher mean cortical density than both cats and small-breed dogs. This difference was observed across the investigated regions and vertebral levels, indicating a relatively more robust cortical structure in toy-breed dogs within the study population. This finding may suggest a higher implant retention potential in toy-breed dogs than in cats, provided adequate implant anchorage is achieved.

Because the DALR in our study showed neither breed-specific differences (as previously reported by Schmidli et al. [2019]) nor interspecific differences, our findings support the assumption proposed by Takahashi et al. (2017) that changes in this parameter may indicate a dens anomaly.

The homogeneous distribution pattern of trabecular microarchitecture observed in cats across segments C2 to C4 could indicate more uniform mechanical stress along the caudal cervical spine. In particular, the lower variability between vertebral bodies suggests adaptive behavior of the trabecular bone within this region. Furthermore, this distribution pattern may reflect species-specific differences in behavior, locomotion, and movement patterns between cats and dogs, which could result in different mechanical loading conditions and consequently influence trabecular bone adaptation. In contrast, toy-breed dogs and small-breed dogs showed greater regional variability in the parameters examined, which could indicate more differentiated local stress conditions within the individual vertebral segments. The lower values in region B in cats could be attributed to reduced mechanical stress in this area. However, whether these differences have functional consequences for structural stability cannot be conclusively assessed from the available data and requires further biomechanical investigation.

No trabecular differences were observed between the evaluated dog breeds within the atlas region. This finding may indicate that the axis plays a more critical role in the development of AAI than the atlas. Alternatively, the absence of significant differences may be attributable to chance. In addition, no dogs with clinically manifest AAI were included in the present study; therefore, disease-specific microarchitectural changes may not have been captured.

These findings suggest that implant placement in cats requires similar caution to that in toy-breed dogs. Implant stability is primarily determined by the contact area with cancellous bone and the overlying cortical bone (42, 45). In dorsal stabilization techniques, postoperative complications such as implant failure and bone fractures have been reported (13, 17), and reduced cortical bone density may increase the risk of intraoperative or postoperative fractures during fixation (41). Accordingly, decreased cortical bone strength should be taken into account when selecting the implant type and surgical technique to avoid overloading the bone. At the same time, the trabecular architecture may contribute overall to implant support.

Nevertheless, factors such as age and duration of clinical signs appear to have a greater influence on treatment outcome than the choice of surgical technique (10). Given the limited surgical options for cats and toy-breed dogs with fragile bone structure, conservative management may represent a viable alternative in selected cases (8). Ultimately, treatment decisions should be based on the patient’s specific clinical condition and biomechanical requirements.

Interestingly, AAI is rarely diagnosed in cats, although the observed structural vulnerability in their bone architecture would theoretically suggest a higher predisposition. One possible explanation is that cats with severe instabilities often die or are euthanized before a clinical diagnosis is made, resulting in the condition being recognized less frequently in clinical practice. In addition, differences in behavior, activity, and injury mechanics between cats and dogs may contribute to the development and clinical manifestation of AAI (3). These species-specific differences may also influence trabecular bone microarchitecture through differences in mechanical loading and should therefore be considered when interpreting the observed microarchitectural differences between cats and dogs.

A limitation of the present study is the age distribution of the animals, as no young individuals were included in the study population. Consequently, only limited conclusions can be drawn regarding younger animals. Both cortical and trabecular bone undergo a gradual age-related decline in function and structure (46), and bone microarchitecture may vary considerably with age. Therefore, age-related differences cannot be fully assessed in this dataset, and the observed findings may have been influenced by age-associated changes in bone properties.

## 5 Conclusion

This study provides the first detailed description of the trabecular and cortical microarchitecture of the first four cervical vertebrae in dogs and cats. The results indicate that trabecular bone structure is largely comparable between toy- and other small-breed dogs, whereas cortical density appears to be more consistent in toy-breed dogs. In addition, toy-breed dogs showed higher cortical density than cats, suggesting potential differences in structural bone characteristics between these species.

Overall, these cervical vertebral characteristics may be relevant for surgical planning and should be considered, where possible, during preoperative evaluation. In agreement with previous studies, a ventral approach is recommended for the surgical management of cervical spine disorders, including AAI, in both species. The present study was conducted in healthy animals, which limits the direct transferability of the findings to pathological conditions. Future studies should therefore include animals with cervical instability or osseous defects and further evaluate potential implant placement sites under clinical conditions.

## Data Availability

The original contributions presented in the study are included in the article/Supplementary material, further inquiries can be directed to the corresponding author.
