# Supplementary material for: Microcomputed tomography examination of the cortical and trabecular structures of the cranial cervical spine in dogs and cats
Source: Front Vet Sci. 2026 Jul 23;13:1888800. doi: 10.3389/fvets.2026.1888800 (PMC13441857; doi:10.3389/fvets.2026.1888800)
Supplement: Supplementary file 1 [file Table_1.DOCX]

Supplementary Material

Supplementary Table 1: Median Values and Corresponding Confidence Intervals of Bone Microarchitecture Parameters in Cervical Vertebra C2, Subdivided by Anatomical Region, Stratified by Breed, Excluding two dogs lacking trabecular structure in the Spinous Process.

| C2 | Parameter | small dogs (n=10) | toy breeds (n=10) |
| --- | --- | --- | --- |
| Dens | BV/TV (%)  BS/BV (mm-1)  Conn.D (mm-3)  DA (dimensionless)  Tb.Sp (mm)  Tb.N (mm-1)  Tb.Th (mm)  DALR (%) | 0.688 (0.591-0.785)*  9.51 (7.46-11.6)*  12.08 (8.94-15.2)*  1.29 (1.24-1.33)†  0.101 (0.0394-0.162)  3.13 (2.89-3.38)  0.214 (0.1864-0.242)*  0.380 (0.369-0.390) | 0.538 (0.441-0.635)*  12.65 (10.61-14.7)*  18.48 (15.34-21.6)*  1.35 (1.30-1.40)†  0.132 (0.0711-0.194)  3.26 (3.02-3.50)*  0.163 (0.1353-0.190)*  0.377 (0.367-0.0.388) |
| Spinous  process | BV/TV (%)  BS/BV (mm-1)  Conn.D (mm-3)  DA (dimensionless)  Tb.Sp (mm)  Tb.N (mm-1)  Tb.Th (mm) | 0.487 (0.389-0.586)  10.15 (8.08-12.2)  7.23 (3.95-10.5)  1.41 (1.36-1.46)  0.227 (0.1647-0.290)  2.34 (2.09-2.59)  0.206 (0.1780-0.234)* | 0.411 (0.313-0.509)  12.34 (10.26-14.4)  9.36 (6.08-12.6)  1.35 (1.30-1.41)  0.249 (0.1868-0.312)  2.40 (2.15-2.65)  0.166 (0.1380-0.194)* |
| C2A | BV/TV (%)  BS/BV (mm-1)  Conn.D (mm-3)  DA (dimensionless)  Tb.Sp (mm)  Tb.N (mm-1)  Tb.Th (mm) | 0.352 (0.255-0.449)  14.96 (12.91-17.0)†  14.23 (11.09-17.4)  1.28 (1.24-1.33)  0.280 (0.2186-0.341)*  2.44 (2.20-2.68)†  0.137 (0.1094-0.164) | 0.255 (0.158-0.352)  17.43 (15.39-19.5)†  13.49 (10.36-16.6)  1.30 (1.25-1.35)  0.373 (0.3117-0.435)*  2.12 (1.87-2.36)†  0.115 (0.0877-0.143) |
| C2B | BV/TV (%)  BS/BV (mm-1)  Conn.D (mm-3)  DA (dimensionless)  Tb.Sp (mm)  Tb.N (mm-1)  Tb.Th (mm) | 0.478 (0.381-0.575)  12.37 (10.32-14.4)  11.93 (8.79-15.1)  1.45 (1.41-1.50)  0.204 (0.1426-0.265)  2.69 (2.45-2.93)  0.166 (0.1385-0.194) | 0.476 (0.379-0.573)  10.57 (8.52-12.6)  10.69 (7.55-13.8)  1.50 (1.45-1.55)  0.211 (0.1500-0.273)  2.66 (2.41-2.90)  0.190 (0.1625-0.218) |
| C2C | BV/TV (%)  BS/BV (mm-1)  Conn.D (mm-3)  DA (dimensionless)  Tb.Sp (mm)  Tb.N (mm-1)  Tb.Th (mm) | 0.456 (0.358-0.553)  12.58 (10.53-14.6)  14.67 (11.53-17.8)  1.13 (1.08-1.18)  0.208 (0.1462-0.269)  2.71 (2.47-2.96)  0.164 (0.1365-0.192) | 0.364 (0.267-0.461)  14.93 (12.88-17.0)  16.33 (13.20-19.5)  1.13 (1.08-1.18)  0.255 (0.1939-0.317)  2.61 (2.37-2.85)  0.136 (0.1088-0.164) |

BV/TV - bone volume fraction; BS/BV - bone surface to volume ratio; Conn.D - connectivity density; DA - degree of anisotropy; Tb.Sp - trabecular separation; Tb.N - trabecular number; Tb.Th - trabecular thickness; DALR - dens-to-axis-length-ratio.
Regions: Dens, Spinous process, cranial corpus (C2A), middle corpus (C2B), caudal corpus (C2C).
Comparisons were performed between small dogs and toy breeds. Significant differences (P < 0.05) are indicated by an asterisk (*), trends (P = 0.05–0.1) by a dagger (†).

Supplementary Table 2: Median Values and Corresponding Confidence Intervals of Bone Microarchitecture Parameters in Cervical Vertebra C2, Subdivided by Anatomical Region, Stratified by Animal Species, Excluding one dog lacking trabecular structure in the Spinous Process.

| C2 | Parameter | toy breeds (n=10) | cats (n=20) |
| --- | --- | --- | --- |
| Dens | BV/TV (%)  BS/BV (mm-1)  Conn.D (mm-3)  DA (dimensionless)  Tb.Sp (mm)  Tb.N (mm-1)  Tb.Th (mm)  DALR (%) | 0.538 (0.441-0.635)*  10.94 (9.62-12.3)*  15.68 (13.75-17.61)*  1.31 (1.28-1.35)*  0.117 (0.0732-0.161)  3.19 (3.03-3.36)*  0.188 (0.1722-0.204)†  0.378 (0.368-0.0.389) | 0.724 (0.6668-0.781)*  9.76 (8.43-11.1)  12.27 (10.34-14.21)*  1.47 (1.44-1.51)*  0.0698 (0.0260-0.113)  3.69 (3.53-3.86)*  0.208 (0.1920-0.224)†  0.387 (0.377-0.398) |
| Spinous  process | BV/TV (%)  BS/BV (mm-1)  Conn.D (mm-3)  DA (dimensionless)  Tb.Sp (mm)  Tb.N (mm-1)  Tb.Th (mm) | 0.456 (0.3979-0.513)*  11.13 (9.78-12.5)*  7.96 (5.95-9.97)*  1.40 (1.36-1.44)  0.2384 (0.1938-0.283)*  2.35 (2.18-2.53)*  0.185 (0.1685-0.201)* | 0.153 (0.0957-0.209)*  17.05 (15.72-18.4)*  4.09 (2.16-6.02)*  1.39 (1.36-1.43)  0.7198 (0.6761-0.763)*  1.23 (1.06-1.40)*  0.119 (0.1028-0.135)* |
| C2A | BV/TV (%)  BS/BV (mm-1)  Conn.D (mm-3)  DA (dimensionless)  Tb.Sp (mm)  Tb.N (mm-1)  Tb.Th (mm) | 0.306 (0.2495-0.363)  16.10 (14.77-17.4)  13.47 (11.53-15.40)  1.29 (1.25-1.33)  0.3204 (0.2767-0.364)*  2.28 (2.12-2.45)*  0.127 (0.1113-0.143) | 0.344 (0.2874-0.401)  15.56 (14.23-16.9)  15.28 (13.35-17.21)  1.31 (1.28-1.35)  0.2564 (0.2127-0.300)*  2.57 (2.40-2.74)*  0.131 (0.1155-0.147) |
| C2B | BV/TV (%)  BS/BV (mm-1)  Conn.D (mm-3)  DA (dimensionless)  Tb.Sp (mm)  Tb.N (mm-1)  Tb.Th (mm) | 0.484 (0.4267-0.540)*  11.88 (10.56-13.2)*  10.31 (8.38-12.24)  1.48 (1.44-1.51)  0.2089 (0.1652-0.253)*  2.67 (2.50-2.83)*  0.171 (0.1548-0.186)* | 0.242 (0.1849-0.299)*  18.04 (16.71-19.4)*  11.58 (9.65-13.52)  1.52 (1.48-1.56)  0.3709 (0.3272-0.415)*  2.11 (1.94-2.28)*  0.111 (0.0956-0.127)* |
| C2C | BV/TV (%)  BS/BV (mm-1)  Conn.D (mm-3)  DA (dimensionless)  Tb.Sp (mm)  Tb.N (mm-1)  Tb.Th (mm) | 0.414 (0.3570-0.471)*  13.66 (12.34-15.0)*  15.43 (13.50-17.37)  1.13 (1.09-1.17)*  0.2311 (0.1873-0.275)  2.66 (2.49-2.83)  0.150 (0.1343-0.166)* | 0.324 (0.2668-0.381)*  16.68 (15.35-18.0)*  17.48 (15.54-19.41)  1.36 (1.32-1.40)*  0.2623 (0.2185-0.306)  2.59 (2.42-2.76)  0.123 (0.1071-0.139)* |

BV/TV - bone volume fraction; BS/BV - bone surface to volume ratio; Conn.D - connectivity density; DA - degree of anisotropy; Tb.Sp - trabecular separation; Tb.N - trabecular number; Tb.Th - trabecular thickness; DALR - dens-to-axis-length-ratio.
Regions: Dens, Spinous process, cranial corpus (C2A), middle corpus (C2B), caudal corpus (C2C).
Comparisons were performed between toy breeds and cats. Significant differences (P < 0.05) are indicated by an asterisk (*), trends (P = 0.05–0.1) by a dagger (†).
